# Supplementary material for: Childhood Obesity Prevention in Africa: A Systematic Review of Intervention Effectiveness and Implementation
Source: Int J Environ Res Public Health. 2019 Apr 4;16(7):1212. doi: 10.3390/ijerph16071212 (PMC6480149; doi:10.3390/ijerph16071212)
Supplement: Supplementary file 1 [file ijerph-16-01212-s001.pdf]

Supplementary table 1. Search terms (including MeSH terms)

Search Term 1:

child* or childhood/ or child growth/ or child nutrition/ or adolescen* or "young person*" or "young people" or teen* or youth* or boy* or girl* or juvenile* or juvenile/ or ″early childhood″ or pre-school or preschool or kindergarten or nursery or nursery school/ or school-age or ″child care″ or childcare or ″primary school″ or primary school/ or ″elementary school″ or ″middle school″ or middle school/ or middle school student/ or ″high school″ or high school/ or high school student/ or p!ediatric* or preadolescen* or pre-adolescen*

Search Term 2:

overweight or obes* or obesity/ or bmi or ″body mass″ or body mass/ or ″body mass index″ or ″body-mass index″ or ″body weight″ or body weight/ or ″body composition″ or weight or ″weight status″ or ″body size″ or fatness or ″body fat″ or adipos* or ″nutritional status″ or nutritional status/

Search Term 3:

"physical* activ*" or physical activity/ or sport* or sport/ or youth sport/ or "physical education" or physical education/ or "physical training" or exercis* or exercise/ or "energy expenditure" or energy expenditure/ or "physical inactivity" or physical inactivity/ or "physical fitness" or "active travel" or sedentary or motor activity/ or physical exertion/ or "physical education and training" or ″physical activity environment″ or fitness or fitness/ or inactivity or ″dietary intake″ or dietary intake/ or ″dietary behavio!r*″ or eating or eating/ or diet* or diet/ or nutrition* or nutrition/ or ″nutrition* intervention*″ or lifestyle* or life-style* or feeding* or feeding behaviour/ or ″sedentary behavio!r*″ or sedentary lifestyle/ or food* or food/ or ″food intake″ or food intake/ or ″food environment″ or meal* or ″dietary diversity″ or ″fruit consumption″ or fruit* or fruit/ or sugar* or sugar/ or sugar intake/ or snack* or ″sugar-sweetened beverage*″ or drink* or ″fast food*″ or fast food/ or ″health* behavio!r*″ or ″unhealthy behavio!r*″ or family-based or community-based or home-based or school-based or parent* or teacher* or ″active lesson*″ or ″school lunch″ or lunchbox or ″lunch box″ or ″school food″ or tuckshop* or vendor* or ″food price*″ or weight-related or ″junk food″ or ″screen time″ or ″television viewing″ or television viewing/ or TV or ″computer use″ or ″portion size*″ or portion size/ or exergame or MVPA

Search Term 4:

Africa/ or Algeria or Angola or Benin or Botswana or ″Burkina Faso″ or Burundi or Cameroon or ″Cape Verde″ or ″Central African Republic″ or Chad or Comoros or Congo or ″Democratic Republic of Congo″ or Djibouti or Egypt or ″Equatorial Guinea″ or Eritrea or Ethiopia or Gabon or Gambia or Ghana or Guinea or ″Guinea Bissau″ or ″Ivory Coast″ or ″Cote d′Ivoire″ or Kenya or Lesotho or Liberia or Libya or Madagascar or Malawi or

Supplementary resource 2. African journals screened based on recommendations

- African Journal for Physical, Health Education, Recreation and Dance
- South African Journal of Sports Medicine
- Journal of Public Health in Africa
- South African Medical Journal
- South African Journal for Research in Sport, Physical Education and Recreation
- African Journal for Physical Activity and Health Sciences
- South African Journal of Clinical Nutrition
- South African Family Practice
- South African Journal of Child Health

Supplementary table 3. Measured outcomes and reported effects of included studies

Study	Measured outcomes of relevance	Reported effects
**DoH Health Promoting Schools**<br><br>Nyawose & Naidoo 2016 [54]	Sports and PA participation (learner questionnaires that have been used in other South African studies and "have been shown to be valid and reliable"), Fitness (Eurofit Physical Fitness Test Battery (Eurofit, 1993)). Height and weight.	*Weight:*<br>I: 41.18±9.04 → 41.09 ±9.84, p=0.003*; C: 41.33 ±7.76 → 42.68 ±7.71, p<0.0005*<br><br>*Sit-and-reach:*<br>I: 33.47±5.55 → 34.10±5.85, p=0.43*; C: 29.74±7.54 → 30.03±7.88, p=0.501<br><br>*Sit-ups:*<br>I: 18.24±4.88 → 19.20±4.87, p=0.007*; C: 17.48±2.68 → 17.94±3.31, p=0.414<br><br>*Shuttle run:*<br>I: 20.55±2.04 → 19.47±4.10, p<0.0005*; C: 22.29±8.58 → 21.35±2.10, p=0.649<br><br>*Plate tapping:*<br>I: 15.33±2.29 → 13.24±1.74, p<0.0005*; C: 14.11±1.98 → 13.52±1.83, p=0.002*<br><br>*Flamingo balance:*<br>I: 12.24±4.76 → 13.36±4.62, p=0.011*; C: 12.45±3.29 → 16.25±5.48, p<0.0005*<br><br>*Standing long jump:*<br>I: 136.10±21.75 → 133.41±19.95, p=0.306; C: 150.47±26.35 → 149.20±20.57, p=0.306<br><br>Prevalence of overweight or obesity 0% in both I and C<br>* p< 0.05

**Gum Marom Kids League (GMKL)**<br><br>Richards et al. 2014[63]	Physical fitness (Cardiorespiratory fitness was measured using the multi-stage fitness test (MFT), Muscle power and strength measured through standing broad jump (SBJ), Anthropometric outcomes (BMI-for-age (BFA) and height-for-age (HFA) z-scores based on 2007 normative values using the WHO AnthroPlus software). Mental health outcomes reported but not extracted here.	*Multi-stage fitness test, km/hr (baseline = BL, follow-up = F-U)*<br>I: Boys BL 11.14, boys F-U 11.46$^{\#}$, girls BL 10.10, girls F-U 10.43$^{\#}$<br>Waitlist: Boys BL 11.10, boys F-U 11.58$^{\#}$<br>C: Boys BL 10.96, boys F-U 11.29$^{\#}$, girls BL 9.70, girls F-U 10.19$^{\#}$<br><br>*Standing broad jump, cm*<br>I: Boys BL 187.55, boys F-U 181.59$^{\#}$, girls BL 166.53, girls F-U 167.52<br>Waitlist: Boys BL 184.21, boys F-U 186.55<br>C: Boys BL 181.80, boys F-U 181.68, girls BL 163.42, girls F-U 163.29<br><br>*BMI-for-age, z-score*<br>I: Boys BL −0.65, boys F-U −0.74, girls BL −0.27, girls F-U −0.32<br>Waitlist: Boys BL −0.64, boys F-U −0.71<br>C: Boys BL −0.66, boys F-U −0.67, girls BL −0.21, girls F-U −0.25$^{\#}$<br><br>$^{\#}$=Statistically significant within-group change (p<0.05)
Harrabi et al. 2010 [48]	Pre-tested self-administered questionnaire for knowledge, behaviours, and intentions about smoking, dietary habits, and physical activity. Anthropometric measurements were also taken (height and weight) but not reported.	*Eating at least 5 fruits and vegetables per day, pre-post:*<br>I: 45.3 → 55.4, p=0.06<br>C: 48.3 → 57.9, p=0.03<br><br>*Practice more than 30 min of physical activity for at least six days a week, pre-post:*<br>I: 17.5 → 35.9, $p<10^{-3}$*<br>C: 27.2 → 36.9, $p<10^{-3}$<br><br>*Percentage change reported as significantly higher by authors

**HealthKick**<br><br>Steyn et al. 2015 [46],<br>De Villiers et al. 2016 [64],<br>Uys et al. 2016 [47]	Dietary diversity score (unquantified 24-h recall), fat consumption, sugar consumption, other components of DDS (unquantified24-h recall). Fitness (Measured using modified Eurofit) and KAB (validated questionnaire). nutritional behaviour (score) (collected via questionnaire which was pilot tested but not validated, anthropometry methods not specified, nor what was primary/secondary)	*Dietary diversity score:*<br>BL to FU2 estimated intervention effect 0.04 (−0.37; 0.46) p=0.826<br><br>*Fat consumption:*<br>BL to FU2 estimated intervention effect −0.03(−0.26; 0.20) p=0.809.<br><br>*Sugar consumption:*<br>BL to FU2 estimated intervention effect −0.27 (−0.68; 0.13) p=0.165<br><br>*Nutritional behaviour score:*<br>BL-FU2 estimated intervention effect .09 (.47, .64) p=0.743<br><br>*Multilevel Model Analysis of Fitness Tests between Intervention and Control Groups, adjusted results 2009-2010*<br><br>Sit-and-Reach (cm): -0.15 (-1.28, 0.97)<br>Sit-ups: 2.17 (1.22, 3.13)*<br>Shuttle Run (sec): 0.85 (-0.89, 2.59)<br>Standing Long Jump (cm): 1.71 (-1.89, 5.30)<br><br>*Multilevel Model Analysis of Fitness Tests between Intervention and Control Groups, adjusted results 2010-2011*<br><br>Sit-and-Reach (cm): -1.29 (-2.43, -0.14)*<br>Sit-ups: 1.62 (0.65, 2.59)*<br>Shuttle Run (sec): 3.32 (1.56, 5.08)*<br>Standing Long Jump (cm): -5.75 (-9.39, -2.11)*

**Healthnutz**<br><br>Draper et al. 2010 [53]	Anthropometric measurements (height and weight), physical fitness. Fitness: Eurofit Fitness Testing protocol, which has been adapted for use in a South African setting. Other questionnaires based on validated questionnaires from similar settings, and pilot-tested.	Pre-post assessments<br><br>*Weight (kg)*<br>I: 35.8 ± 8.6 → 37.1 ± 8.7; C: 36.9 ± 8.4 → 37.1 ± 8.4<br>P-value for group x time interaction p<0.005<br><br>*Sit and reach*<br>I: 14.6 ± 5.9 → 19 ± 6.8; C: 24.5 ± 16 → 14 ± 9.7<br>P-value for group x time interaction p<0.001<br><br>*Sit ups*<br>I: 16 ± 6 → 17.8 ± 6.1; C: 15.2 ± 5.4 → 15.5 ± 5.1<br>P-value for group x time interaction p<0.02<br><br>*Shuttle run*<br>I: 48.5 ± 5.1 → 46.2 ± 4.6; C: 47.2 ± 4.6 → 48.6 ± 5.5<br>P-value for group x time interaction p<0.0001<br><br>*Long jump*<br>I: 124.6 ± 25.7 → 134.3 ± 25.7; C: 120.4 ± 18.8 → 135 ± 19.7<br>P-value for group x time interaction p=0.135<br><br>*Ball throw*<br>I: 22 ± 7.2 → 23.1 ± 7.5; C: 21.7 ± 6.2 → 21.8 ± 6.9<br>P-value for group x time interaction p=0.106

Hochfeld et al. 2016 [52]	Anthropometric measurements (height, weight, BMI using standard protocols)	Severely stunted -4.7%,<br>Stunted 0.3%,<br>Not stunted 4.3%,<br>Severely overweight -4.3%,<br>Overweight -3.1%,<br>Within BMI guidelines for age 10%,<br>Wasted -0.7%,<br>Severely wasted -1.9%.

Kebaili et al. 2014 [65]	Nutrition-related behaviours. A pre-tested self-administered questionnaire was used to collect these data.	Pre-post changes:<br><br>*"Ideal" breakfast intake*<br>I: 4.4% → 10.5%, $p < 10^{-3}$; C: 4.5% → 2.8%, p=0.44<br><br>*Daily breakfast intake*<br>I: 58.2% → 67.5%, $p < 10^{-3}$; C: 53.5% → 53.2%, p=0.883<br><br>*Daily dairy products intake*<br>I: 61.3% → 74.4%, $p < 10^{-3}$; C: 51.6% → 57.1%, p=0.001<br><br>*FVC of five or more times every*<br>I: 29.3% → 31.1%, p=0.683; C: 25.3% → 34.3%, p=0.38<br><br>*Snacking at the evening*<br>I: 59.4% → 52.1%, $p < 10^{-3}$; C: 62.1% → 95.1%, p=0.087<br><br>*Soft drink intake every day*<br>I: 22.6% → 18.8%, p=0.003; C: 22% → 20.5%, p=0.319<br><br>*Fast food intake (three or more times/week)*<br>I: 42.5% → 30.9%, $p < 10^{-3}$; C: 40.5% → 41.2%, p=0.765

Maatoug et al. 2015 [59]	Eating habits, physical activity, screen time. All reported by parents, not reported whether instruments used were validated.	*Dietary behaviour – nibbling (prevalence, %)*<br>I:<br>Executive mother: 75.4 → 61.5, p=0.01<br>Housewife/worker mother: 78.7 → 71.8, p=0.17<br>Executive father: 76.3 → 63.3, p=0.01<br>Worker father: 79.3 → 70.4, p=0.09<br>C:<br>Executive mother: 85.7 → 74.1, p=0.08<br>Housewife/worker mother: 83.5 → 74.7, p=0.04<br>Executive father: 86.1 → 76.7, p=0.08<br>Worker father: 83.5 → 72.9, p=0.05<br><br>*Dietary behaviour – balanced eating habits (prevalence, %)*<br>I:<br>Executive mother: 19.6 → 31.1, p=0.002<br>Housewife/worker mother: 13.9 → 21.5, p=0.09<br>Executive father: 17.8 → 31.1, p=0.007<br>Worker father: 14.7 → 21, p=0.19<br>C:<br>Executive mother: 12.2 → 15.5, p=0.59<br>Housewife/worker mother: 7.9 → 15.1, p=0.05<br>Executive father: 10.7 → 15.1, p=0.36<br>Worker father: 8.3 → 15.3, p=0.01<br><br>*Practice of physical activity outdoors the kindergarten (prevalence, %)*<br>I:<br>Executive mother: 70.4 → 76.1, p=0.28<br>Housewife/worker mother: 75.4 → 71.9, p=0.51<br>Executive father: 69.3 → 75.3, p=0.22<br>Worker father: 76.5 → 72.5, p=0.43

		C:<br>Executive mother: 66.3 → 58.6, p=0.37<br>Housewife/worker mother: 64.4 → 65.3, p=0.87<br>Executive father: 62.6 → 68.6, p=0.35<br>Worker father: 66.9 → 58.6, p=0.17<br><br>*Spend less than 2 hours per day in screen viewing (prevalence, %)*<br>I:<br>Executive mother: 75.4 → 89, p=0.002<br>Housewife/worker mother: 79.2 → 90.7, p=0.005<br>Executive father: 71.6 → 89.3, p=$<10^{-3}$<br>Worker father: 83.2 → 90.3, p=0.09<br>C:<br>Executive mother: 70.8 → 69.1, p=0.9<br>Housewife/worker mother: 67.1 → 75.2, p=0.11<br>Executive father: 66.9 → 70.7, p=0.5<br>Worker father: 70.3 → 75.4, p=0.35<br><br>*Going to the kindergarten on foot (prevalence, %)*<br>I:<br>Executive mother: 22.8 → 24.5, p=0.72<br>Housewife/worker mother: 49.6 → 42.7, p=0.22<br>Executive father: 28.0 → 32.6, p=0.41<br>Worker father: 43.9 → 35.2, p=0.15<br>C:<br>Executive mother: 52.1 → 39.7, p=0.09<br>Housewife/worker mother: 74.2 → 76.2, p=0.69<br>Executive father: 56.2 → 53.5, p=0.75<br>Worker father: 75.4 → 73.9, p=0.76

**"Masikhusele iKamva Lethu" ("Let Us Protect Our Future.")**<br>Jemmott et al. [49]	Dietary behaviour: Fruit and vegetable consumption over past 30 days (self-report using 7-item food frequency questionnaire developed by the National Cancer Institute, no mention of context-specific validation). Physical activity: Self-reported PA over past 7 days (CDC-developed 3 items, no mention of whether these were validated for the context). The authors report measures to increase validity of self-reported behaviour but not clear if instruments validated.	*Met 5-a-Day guideline in the past 30 days, adjusted odds ratio:*<br>1.30 (95% CI: 1.07, 1.58), p=0.008.<br><br>*Servings of fruit per day in the past 30 days, adjusted odds ratio:*<br>0.54 (95% CI: 0.18, 0.90) p=0.003.<br><br>*Servings of vegetables per day in the past 30 days, adjusted odds ratio:*<br>0.77 (95% CI: 0.38, 1.16) p=0.0001.<br><br>*Met physical activity guideline in past 7 days, adjusted odds ratio:*<br>1.56 (95% CI: 1.29, 1.89) p<0.0001.<br><br>*Days intensive cardiovascular physical activity in past 7 days, adjusted odds ratio:* 0.44 (95% CI: 0.27, 0.60) p<0.0001.<br><br>*Days moderate cardiovascular physical activity in past 7 days, adjusted odds ratio:* 0.67 (95% CI: 0.47, 0.86) p<0.0001.<br><br>*Days strength-building physical activity in past 7 days, adjusted odds ratio:*<br>0.35 (95% CI: 0.15, 0.56) p=0.0006.

**Nutrition and Physical Activity (NAP) Pilot**<br><br>Naidoo et al. 2009 [62]	Physical activity (self-report), practices of learners towards PA (learner questionnaire, not reported whether validated).	*Increase in the number of learners performing chores around the house*<br>10% ($p>0.05$)<br><br>*Proportion of learners who did exercise 'more than 5 times' per week*<br>20% → 43%<br><br>*Increase in learners participating in physical activity 'more than 5 times' per week after school*<br>35% → 55% ($p<0.05$)<br><br>*Proportion of learners who 'do not participate'*<br>7% → 2% ($p<0.05$)<br><br>*Sit-and-reach test (cm)*<br>Boys, pre-post: 29.11±6.05 → 29.38±6.45<br>Girls, pre-post: 30.73±6.52 → 31.62±6.89<br><br>*Sit-ups*<br>Boys, pre-post: 18±3 → 20±4*<br>Girls, pre-post: 15±4 → 16±4*<br><br>*Standing broad jump (m)*<br>Boys, pre-post: 1.50±0.21 → 1.60±0.21 Girls, pre-post: 1.30±0.17 → 1.30±0.19<br><br>*BMI ($kg.m^{-2}$)*<br>Boys, pre-post: 19.15±0.52 → 19.95±0.63 Girls, pre-post: 19.94±0.37 → 20.59±0.38

**Nutrition and Physical Activity (NAP)**<br><br>Naidoo & Coopoo 2012 [55]	Physical activity (self-reported, learner questionnaire) and fitness (Eurofit Physical Fitness Test Battery, 1993). Height and weight.	Pre-post changes:<br><br>*Sit-and-Reach (cm)*<br>I: 28.97± 6.31 → 35.07**±9.47<br>C: 30.30± 5.54 → 33.53**±6.80<br><br>*Sit-ups*<br>I: 13.37±4.21 → 16.40**±5.44<br>C: 13.23±3.98 → 15.32**±3.54<br><br>*5m Shuttle Run (seconds)*<br>I: 47.53±5.40 → 46.44*±7.23<br>C: 50.49±4.02 → 49.34±4.00<br><br>*Standing Broad Jump (m)*<br>I: 1.50±0.26 → 1.65**±0.38<br>C: 1.50*±0.21 → 1.55*±0.24<br><br>*Body Mass Index (BMI)*<br>I: 18.90±0.33 → 19.60±0.30<br>C: 18.90±0.27 → 19.15±0.28

**PLAY**<br>Naude et al. 2008 [66], Lennox & Pienaar 2013 [61]	Body composition. BMI measured without specifying methods, and body fat % measured using Bod Pod, and triceps (TSKF) and subscapular (SSKF) skinfolds.<br>Physical activity (24-hour recall questionnaire) for a weekday and one day during the weekend, energy expenditure (accelerometry data from six students, and analysed using Actical software), aerobic capacity ("The Bleep test"). Only PA and anthropometric outcomes extracted here.	Post assessment measurements by group<br><br>*BMI, girls:*<br>High attendance: 20.39±0.12<br>Medium attendance: 20.34±0.12<br>Control: 20.32±0.14<br>p=0.92<br><br>*Waist circumference, girls:*<br>High attendance: 63.85±0.30<br>Medium attendance: 63.96±0.30<br>Control: 62.49±0.38<br>p<0.001*<br><br>*Body fat %, girls:*<br>High attendance: 27.00±0.50<br>Medium attendance: 28.01±0.52<br>Control: 29.35±0.85<br>p=0.05<br><br>*Skinfolds, girls:*<br>High attendance: 28.97±0.79<br>Medium attendance: 27.43±0.77<br>Control: 33.78±0.97<br>p<0.001*<br><br>*BMI, boys:*<br>High attendance: 18.55±0.12<br>Medium attendance: 18.23±0.07<br>Control: 18.19±0.13

		p=0.05<br><br>*Waist circumference, boys:*<br>High attendance: 62.56±0.40<br>Medium attendance: 63.12±0.25<br>Control: 62.53±0.44<br>p=0.6<br><br>*Body fat %, boys:*<br>High attendance: 17.65±0.83<br>Medium attendance: 16.75±0.54<br>Control: 19.94±1.11<br>p=0.04*<br><br>*Skinfolds, boys:*<br>High attendance: 14.39±0.54<br>Medium attendance: 14.85±0.34<br>Control: 17.44±0.60<br>p<0.001*<br><br>*Week METs/16h*<br>1 High attendance pre-post: 79.69 → 81.86<br>2 Medium attendance pre-post: 81.43 → 82.05<br>3 Low attendance pre-post: 83.04 → 82.92<br>Control pre-post: 84.12 → 84.75<br><br>*Week PA*<br>1 pre-post: 2.0 → 2.0<br>2 pre-post: 2.2 → 2.1<br>3 pre-post: 2.3 → 2.2

		C pre-post: 1.92 → 1.94<br><br>*Week TV time*<br>1 pre-post: 1.7 → 2.7*<br>2 pre-post: 1.7 → 2.3*<br>3 pre-post: 1.9 → 2.4*<br>C pre-post: 2.44 → 2.68<br><br>*Bleep test*<br>1 pre-post: 4.6 → 4.5<br>2 pre-post: 5.5 → 5.0<br>3 pre-post: 5.5 → 5.09<br>C pre-post: 4.45 → 4.07<br><br>*Weekend METs/16h*<br>1 pre-post: 74.32 → 75.08<br>2 pre-post: 78.66 → 76.75<br>3 pre-post: 77.37 → 75.85<br>C pre-post: 73.40 → 77.80<br><br>*Weekend PA*<br>1 pre-post: 2.4 → 2.0*<br>2 pre-post: 2.2 → 2.0<br>3 pre-post: 2.1 → 2.3<br>C pre-post: 1.8 → 1.66<br><br>*Weekend TV time*<br>1 pre-post: 2.5 → 3.7*<br>2 pre-post: 3.2 → 3.7<br>3 pre-post: 3.1 → 3.7*

		C pre-post: 3.39 → 4.01<br><br>*p<0.05

**"Schools in Health"**<br><br>Maatoug et al. 2015 [60]	Overweight/obesity (standard anthropometric measurements), physical activity and dietary behaviour (standardised, pretested questionnaire)	*Recommended amount of fruit and vegetables consumed:*<br>I: 30.0 → 33.2, p=0.03; C: 40.2 → 35.0, p=0.001<br><br>*Consumption of fried food (rarely or never):*<br>I: 7.5 → 7.8, p=0.64; C: 7.0 → 7.0, p=0.99<br><br>*Consumption of fast-food (never in past week):*<br>I: 29.8 → 28.4, p=0.33; C: 40.6 → 39.3, p=0.43<br><br>*Recommended physical activity:*<br>I: 29.1 → 25.5, p=0.01; C: 21.1 → 21.2, p=0.89<br><br>*Walk or bike to school:*<br>I: 72.2 → 74.0, p=0.19; C: 79.4 → 71.1, p<.001<br><br>*Sedentary time on school day (>2 h):*<br>I: 38.1 → 40.1, p=0.19; C: 34.3 → 35.4, p=0.48<br><br>*Sedentary time on Sunday (>2 h):*<br>I: 64.1 → 65.1, p=0.54; C: 65.3 → 60.1, p<.001<br><br>*Prevalence of normal weight:*<br>I: 72.4% → 75.5%, p=0.03; C: 80.0% → 77.0%, p=0.02<br><br>*Prevalence of overweight:*<br>I: 20.6% → 18.0%, p=0.03; C: 15.5% → 16.1%, p=0.58<br><br>*Prevalence of obesity:*<br>I: 7.0% → 6.5%, p=0.51; C: 4.5% → 6.9%, p<0.001

		Intervention effect on risk of overweight/obesity:<br>I: 0.84 (0.73–0.97) p=0.02; C: 1.13 (0.97–1.32) p=0.12
Walter 2014 [56]	Physical activity (objectively measured using Actigraph accelerometry).	*PA change from pre to post assessment:*<br>Total METs 0.10 ± 0.22<br>Sedentary -16.90 ± 39.89<br>Light 10.45 ± 30.12<br>MVPA 6.46 ± 16.64<br><br>*Sedentary time, pre-post:*<br>66% of the school day (237 minutes) → 61% (220 minutes)<br>(t=-3.77, p=0.0005, d=0.42)<br><br>*MVPA time, pre-post:*<br>36 minutes → 42 minutes<br>(t=3.45, p=0.001, d=0.39)

C = control group; I = intervention group; NR = not reported; PA = physical activity; PE = physical education
